# Time-dependent specific molecular signatures of inflammation and remodelling are associated with trimethylamine-N-oxide (TMAO)-induced endothelial cell dysfunction

**DOI:** 10.1038/s41598-023-46820-7

**Published:** 2023-11-20

**Authors:** Meyammai Shanmugham, Arun George Devasia, Yu Ling Chin, Kang Hao Cheong, Eng Shi Ong, Sophie Bellanger, Adaikalavan Ramasamy, Chen Huei Leo

**Affiliations:** 1 Science, Math & Technology, Singapore University of Technology & Design, 8 Somapah Road, Singapore, 487372 Republic of Singapore; 2 A*STAR Skin Research Labs (A*SRL), Agency for Science, Technology and Research (A*STAR), 8A Biomedical Grove, #06-06 Immunos, Singapore, 138648 Republic of Singapore; 3 Genome Institute of Singapore (GIS), Agency for Science Technology and Research (A*STAR), 60 Biopolis Street, Genome, Singapore, 138672 Republic of Singapore

**Keywords:** Cardiology, Molecular medicine

## Abstract

Endothelial dysfunction is a critical initiating factor contributing to cardiovascular diseases, involving the gut microbiome-derived metabolite trimethylamine N-oxide (TMAO). This study aims to clarify the time-dependent molecular pathways by which TMAO mediates endothelial dysfunction through transcriptomics and metabolomics analyses in human microvascular endothelial cells (HMEC-1). Cell viability and reactive oxygen species (ROS) generation were also evaluated. TMAO treatment for either 24H or 48H induces reduced cell viability and enhanced oxidative stress. Interestingly, the molecular signatures were distinct between the two time-points. Specifically, few Gene Ontology biological processes (BPs) and Kyoto Encyclopedia of Genes and Genomes (KEGG) pathways were modulated after a short (24H) compared to a long (48H) treatment. However, the KEGG signalling pathways namely “tumour necrosis factor (TNF)” and “cytokine-cytokine receptor interaction” were downregulated at 24H but activated at 48H. In addition, at 48H, BPs linked to inflammatory phenotypes were activated (confirming KEGG results), while BPs linked to extracellular matrix (ECM) structural organisation, endothelial cell proliferation, and collagen metabolism were repressed. Lastly, metabolic profiling showed that arachidonic acid, prostaglandins, and palmitic acid were enriched at 48H. This study demonstrates that TMAO induces distinct time-dependent molecular signatures involving inflammation and remodelling pathways, while pathways such as oxidative stress are also modulated, but in a non-time-dependent manner.

## Introduction

Endothelial dysfunction is closely associated with increased risk of cardiovascular diseases (CVDs), a leading cause of death worldwide^1^, while interruption in the progression of endothelium dysfunction reduces the occurrence of CVDs^2–4^. Factors contributing to CVDs include hypertension, diabetes mellitus, dyslipidaemia, obesity, age, as well as lifestyle practices such as alcohol consumption, physical inactivity, and diet^5^. In fact, dietary risk factors are key to CVD occurrence, before other common risk factors such as blood pressure or obesity, because they heighten the risk of inflammatory and apoptotic phenotypes that causes endothelial dysfunction^6,7^. In particular, dietary nutrients such as choline, betaine, lecithin, and L-carnitine found in red meat, fish, and egg yolk, are precursors of trimethylamine-N-oxide (TMAO), recently found to enhance the risk of CVDs^8,9^. These dietary nutrients are first converted into trimethylamine (TMA) by gut microbes^10^, before being oxidized into TMAO by flavin monooxygenases (FMOs) enzymes in the liver^11^.

Recent studies identified TMAO as a fundamental factor that drives inflammation and expression of adhesion molecules (2 mechanisms involved in endothelial dysfunction) through ROS modulation *in vitr*o, and high levels of circulating TMAO activate endothelial dysfunction in humans^12^ and are associated with diastolic issues^13^, heart failure^14^, atherosclerotic plaque deposition^15^, peripheral artery disease^16^, and diabetes^17–19^. However, several studies reported controversial results regarding the effects of TMAO on endothelial cells (ECs). For instance, TMAO is not cytotoxic for bovine aortic endothelial cells (BAE-1), where it does not cause any ROS generation^20^. Furthermore, the concentration of TMAO precursors, but not TMAO itself, is linked to metabolic syndrome, cardiovascular phenotypes, and inflammatory biomarkers in humans^21^. Hence, the role of TMAO as a cause of endothelial dysfunction and its potential application as a biomarker both remain unclear, and the mechanisms leading to the diseased state are debatable^22,23^. The discrepancies between studies on TMAO-induced endothelial dysfunction may be attributed to different cell types and animal models used, including different metabolic backgrounds, as well as to distinct TMAO treatment concentrations and durations. While most of the studies evaluated the endpoint impact of TMAO, little is known about the time-dependent TMAO-induced gene transcription profiles in endothelial cells (ECs). Moreover, most studies were performed in human umbilical vein endothelial cells (HUVEC), and human aortic endothelial cells (HAEC) where TMAO has been shown to cause endothelial dysfunction, a risk factor of macrovascular diseases. Hence, our study aims to investigate the impact of TMAO on microvascular endothelial cells which, is less understood. In this study, we used global approaches to investigate the chronological sequence of molecular signatures associated with TMAO-induced endothelial dysfunction in microvascular endothelial cells.

## Results

### TMAO induces oxidative stress and reduces endothelial cell viability

**Figure 1:**
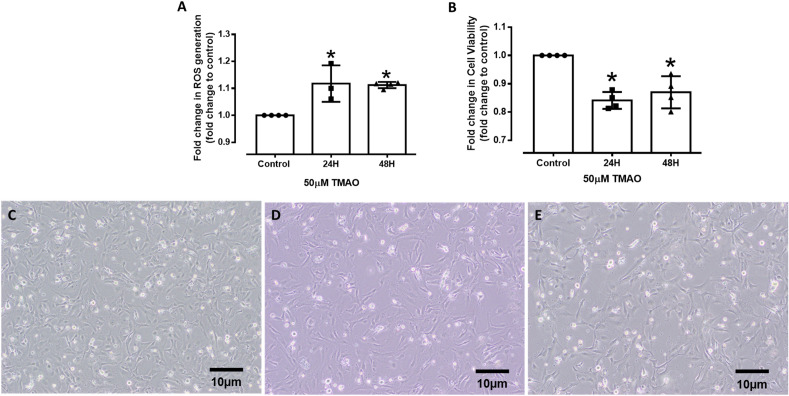
TMAO activates ROS production and lowers cell viability in HMEC-1. (**A**) Quantification of ROS. (**B**) Quantification of cell viability. Values are expressed as mean ± SEM; the number of experiments per group is shown by individual data points. (**C**) Brightfield microscopy images of untreated control cells, (**D**) 24H of 50 µM TMAO treatment, and (**E**) 48H of 50 µM TMAO treatment. *Significantly different from control (P < 0.05, One-way ANOVA, Dunnett’s test).

In most individuals, half of the TMAO produced is not metabolised and is excreted within 24H without any changes via urine, sweat, and breath. However, the remaining 50% may not be excreted from the body and may remain in systemic circulation^13^. Hence, based on the reported metabolism of TMAO in individuals, our time-dependent analysis was performed between the two crucial timepoints of 24H and 48H. To investigate the induction of oxidative stress in HMEC-1 by TMAO, ROS production levels were measured in 50 µM TMAO-treated cells after 24H and 48H (Fig. 1A). TMAO treatment induced a small (~ 10%) but significant increase in ROS levels at both time-points when compared to the control. In addition, higher concentrations of TMAO (150 and 250 µM) also significantly increased ROS levels for both time-points (Supplementary Fig. 1A-B). As oxidative stress is associated with reduced cell death or reduced cell growth, we explored the time-dependent effects of TMAO on HMEC-1 cell viability. When quantified using PrestoBlue, cell viability was significantly reduced at both 24H and 48H, indicating that TMAO (50 µM) lowered proliferation and/or caused cell death (Fig. 1B). Indeed, cell counting from the respective microscopy images also demonstrated that the cell density was significantly reduced at both 24H (76 ± 7%, *n* = 4, *p* < 0.05) and 48H (52 ± 4%, n = 4, *p* < 0.05) after TMAO treatment when compared to the control (100 ± 0%, n = 4) (Fig. 1C-E). Similarly, a reduction in cell viability was observed with higher concentrations of TMAO (150 and 250 µM) at both time-points (Supplementary Fig. 1C-D). While all the tested TMAO concentrations observed reduced the number of viable cells compared to the control, subsequent experiments were conducted using 50 µM TMAO as this concentration was reported to be physiologically relevant to disease conditions in clinical studies^17,24^. Furthermore, given that ROS levels and cell viability between controls were comparable for 24H and 48H treatments (data not shown), controls from the two different timepoints (24H and 48H) were pooled for subsequent RNA sequencing and PCR analysis.

### Transcriptomic profiling (differentially expressed gene analysis)

After 24H of TMAO treatment, only 9 genes were found significantly downregulated (none was found upregulated), while 214 genes were significantly modulated (90 upregulated and 124 downregulated) after 48H of TMAO treatment (Supplementary Figs. 2A and 3). Interestingly, only one gene, dual specificity phosphatase 1 (DUSP1), was common for 24H and 48H, but surprisingly, it was downregulated at 24H (0.66-fold change; FDR = 9.0 × 10^–9^), while upregulated at 48H (1.5-fold change; FDR = 1.1 × 10^–11^) (Supplementary Fig. 2B). The protein encoded by DUSP1 is a phosphatase that has dual specificity for amino acids such as tyrosine and threonine. DUSP1 is known to play a significant role in the human cellular response to environmental stress and negative regulation of cell proliferation, via dephosphorylation of the mitogen-activated protein kinase (MAPK) MAPK1/ERK2^25^. The promoter region of the DUSP1 gene comprises various binding sites for several transcription factors, such as the activator protein 1 (AP-1), nuclear factor-κB (NF-κB), cAMP response element-binding protein, and the glucocorticoid receptor. Therefore, DUSP1 can be activated by various conditions such as inflammation, altered cellular metabolism and the production of excess glucocorticoids during stress responses^26^, all of which have been shown to be triggered by TMAO^12^.

### Enriched gene ontology biological processes (GO BPs)

**Figure 2:**
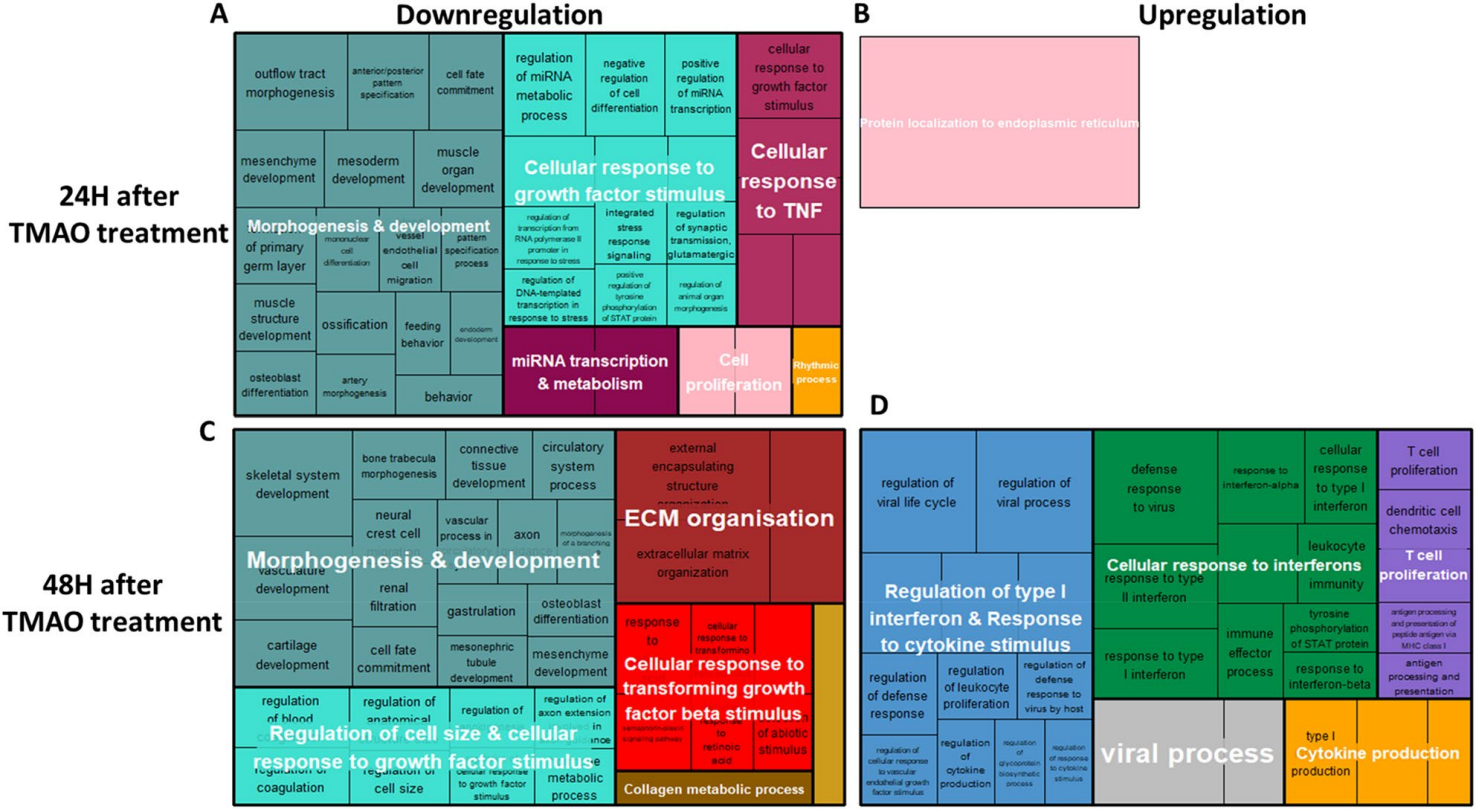
Treemap summary of the GO biological processes enriched after 24H or 48H of TMAO treatment. (**A**) Pathways downregulated after 24H of TMAO treatment. (**B**) Pathways upregulated after 24H of TMAO treatment. (**C**) Pathways downregulated after 48H of TMAO treatment. (**D**) Pathways upregulated after 48H of TMAO treatment.

GO BP analysis after Gene set enrichment analysis (GSEA) allowed the identification of biologically meaningful patterns that were not readily distinguished using the differentially expressed gene (DEG) analysis. Genes involved in BPs related to “morphogenesis & development” as well as “cellular response to growth factor stimulus” were downregulated after both 24H and 48H of TMAO treatment (Fig. 2A and C). In addition, at 24H, we also observed repression of genes involved in “cellular response to TNF”, “cell proliferation”, “miRNA transcription and metabolism” and "rhythmic processes”, while genes involved in “ECM organisation”, “cellular response to transforming growth factor beta 1 (TGF-β)” and “collagen metabolic process” were specifically downregulated at 48H. Conversely, there was a positive enrichment of genes related to “protein localisation to endoplasmic reticulum” at 24H (Fig. 2B), while at 48H, genes involved in “response of type I interferon and cytokine stimulus”, “cellular response to interferons”, “cytokine production”, T cell proliferation” and viral process” were upregulated (Fig. 2D), suggesting strong inflammatory phenotypes specifically at 48H.

### Enriched Kyoto encyclopedia of genes and genomes (KEGG) pathways

**Figure 3:**
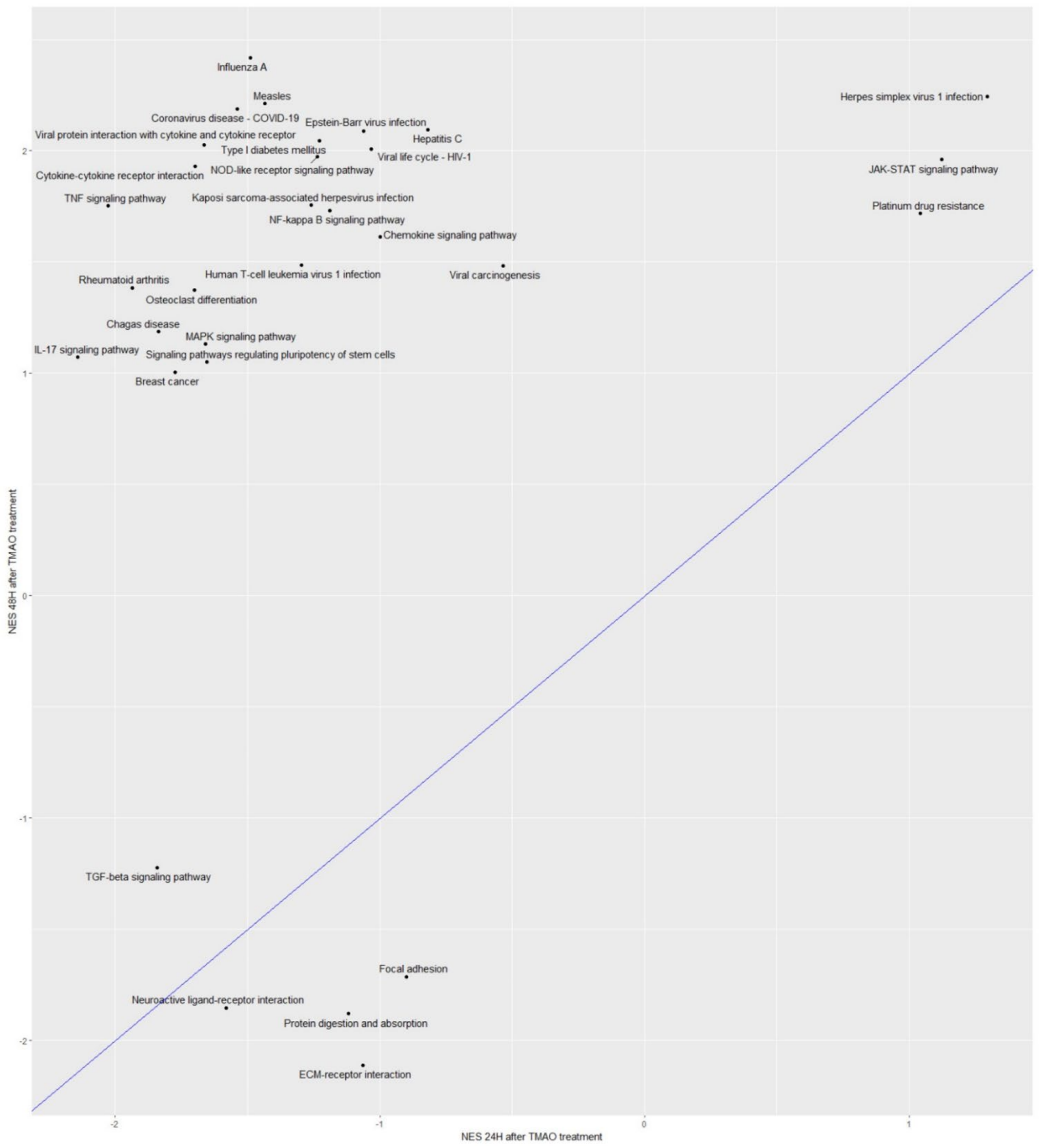
GSEA showing enriched KEGG pathways in HMEC-1 after 24H and 48H of TMAO treatment. The dots shown on the plot refer to normalized enrichment scores (NES) corresponding to highly enriched KEGG pathways modulated by 50 µM of TMAO treatment for 24H (x-axis) or 48H (y-axis). A line of identity (y = x) is shown in blue to indicate the correlation of the pathways in both datasets.

**Figure 4:**
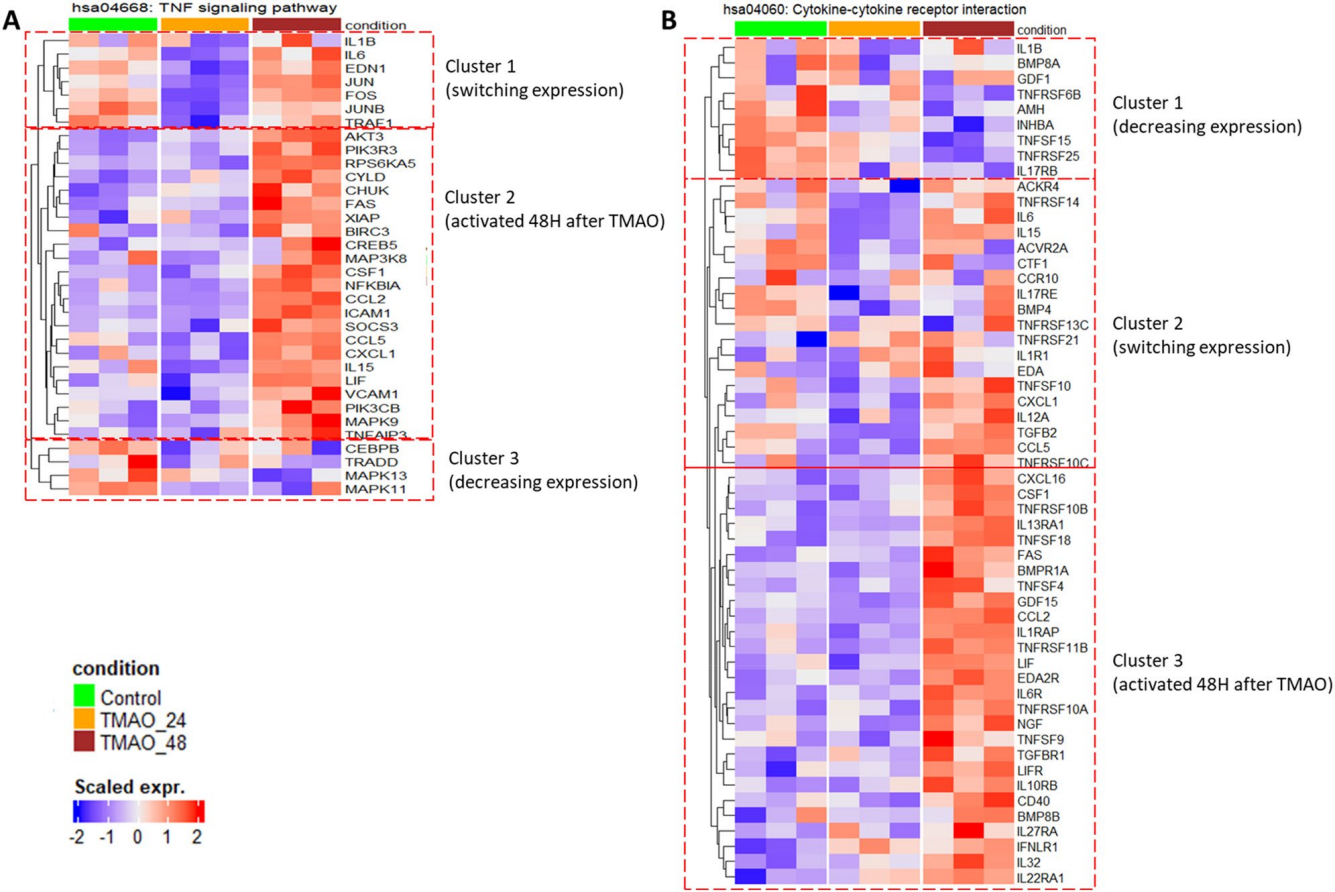
Heatmap of the leading-edge genes identified in two selected pathways (TNF signalling pathway and cytokine-cytokine receptor interaction) through KEGG after GSEA. (**A**) TNF signalling pathway (hsa04668) gene modulation after 24H and 48H of TMAO treatment. (**B**) Cytokine-cytokine receptor interaction (hsa04060) gene modulation after 24H and 48H of TMAO treatment. The scaled expressions of the genes are shown.

KEGG analysis after GSEA confirmed certain findings from GO BP (Fig. 3). Indeed, both time-points showed several similar downregulated pathways (e.g., “focal adhesion” and “ECM receptor interaction”, also found with GO as “collagen metabolic process” and “ECM organisation”), while only the 48H results showed genes activated. Interestingly, the “TNF signalling” (confirmed here to be associated with repressed genes at 24H as found with GO BP), “cytokine-cytokine receptor interaction” and “NOD-like receptor” pathways were identified as positively enriched at 48H (Fig. 3). The heatmaps of the leading-edge genes at 24H and 48H for the 2 first pathways are shown on Fig. 4A/Supplementary Fig. 4A-B and Fig. 4B/Supplementary Fig. 5A-B, respectively. For the TNF signalling pathway (Fig. 4A/Supplementary Fig. 4A-B), cluster 1 genes involving AP-1 (Jun/Fos) signalling and pro-inflammatory interleukins (e.g., interleukin 6 (IL-6)), as well as the endothelin 1 (EDN1) vasoconstrictor, underwent switching in expression from repressed at the early time-point to activated at the late time-point. Cluster 2 genes, mainly inflammatory cytokines, cell adhesion, and leukocyte recruitment molecules, were activated at 48H, while they were moderately low at 24H compared to the control. Gene cluster 3, which showed decreased expression at both 24H and 48H, includes genes involved in MAPK pathways. Expression patterns of “cytokine-cytokine receptor interaction” (Fig. 4, Supplementary Fig. 5A-B) indicate that cluster 1 genes undergo decreased expression at both 24H and 48H. Genes demonstrating a switching expression from repressed at 24H to activated at 48H are grouped in cluster 2 and involve mainly pro-inflammatory cytokines. Cluster 3, corresponding to genes activated at 48H only, shows genes related to the production of inflammatory mediators, cell death and apoptosis, and leukocyte adhesion to ECs.

### TMAO activates endothelial inflammation and cell adhesion after 48H of treatment

**Figure 5:**
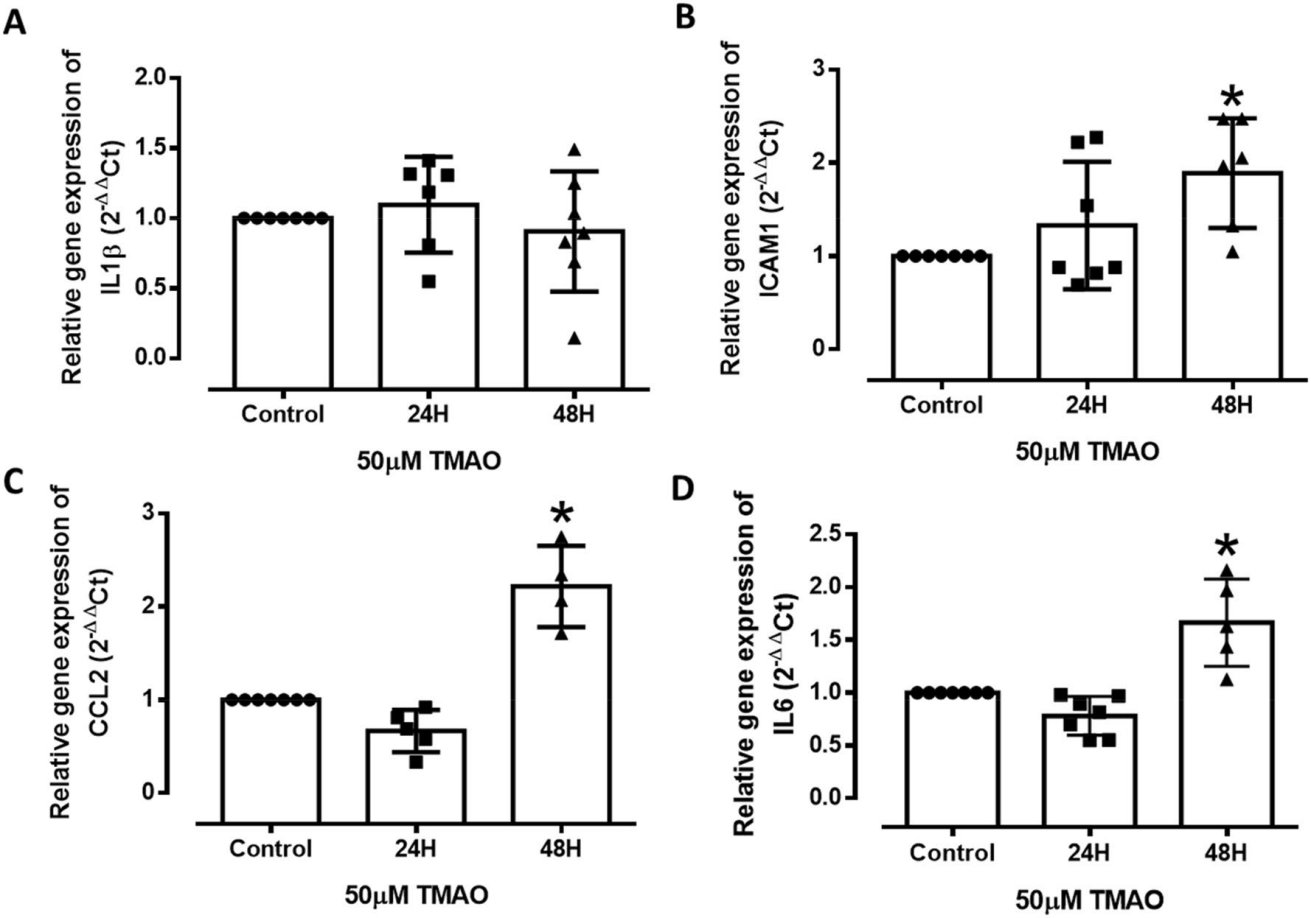
Effect of TMAO on the expression of inflammatory markers in HMEC-1. (**A**-**D**) Relative mRNA expression of IL-1β, ICAM1, CCL2, and IL-6 after 24H and 48H of TMAO treatment compared to untreated cells (Control). Values are expressed as mean ± SEM; the number of experiments per group is shown by individual data points. *Significantly different from control (*P* < 0.05, one-way ANOVA, Dunnett’s test).

As the inflammation and adhesion pathways were recurrently activated in the global analyses, relative gene expression of inflammatory cytokines and adhesion markers, namely IL-6, interleukin 1 beta (IL-1β), intracellular adhesion molecule 1 (ICAM1), and chemoattractant protein 1 (CCL2), were analysed using real-time quantitative polymerase chain reaction (RT-qPCR) at both time-points. IL-1β gene expression did not show any significant changes at both 24H and 48H with levels comparable to the control in both gene sequencing and by qPCR (Fig. 5A). In contrast, a significant upregulation of ICAM1, CCL2, and IL-6 was observed at 48H specifically (Fig. 5B-D), confirming the global analyses. Taken together, these findings correlate with our global analyses indicating more pronounced inflammation and adhesion phenotypes after 48H than after 24H of TMAO treatment.

### TMAO induces endothelial cell remodelling

**Figure 6:**
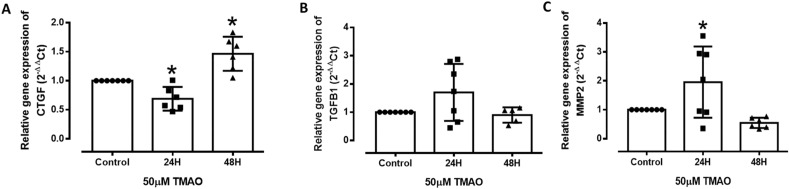
TMAO differentially modulates endothelial cell remodelling in HMEC-1 at 24H and 48H. (**A**-**C**) Relative mRNA expression of CTGF, TGF-β1 and MMP2 after 24H and 48H of TMAO treatment compared to Control. Values are expressed as mean ± SEM; the number of experiments per group is shown by individual data points. *Significantly different from control (*P* < 0.05, One-way ANOVA, Dunnett’s test).

Validation of genes from the GSEA associated with markers of ECM showed that connective tissue growth factor (CTGF) (a central tissue modelling regulator also known to be involved in vascularisation) was significantly downregulated after 24H in both global and qPCR analyses but activated after 48H (Fig. 6A) in the qPCR analysis. In contrast, TGF-β1 (known to regulate ECM gene expression) showed no significant changes at 24H but a decrease at 48H (Fig. 6B) in the global analysis. While matrix metallopeptidase 2 (MMP2) gene expression was significantly activated after 24H qPCR analysis, it showed a significant decrease at 48H in the global analysis (Fig. 6C). The results suggest that ECM remodelling activated by MMP2 is triggered first, while ECM modifications regulated by CTGF and TGF-β1 occur at a later stage.

### Metabolite profiling by LC/MS

**Figure 7:**
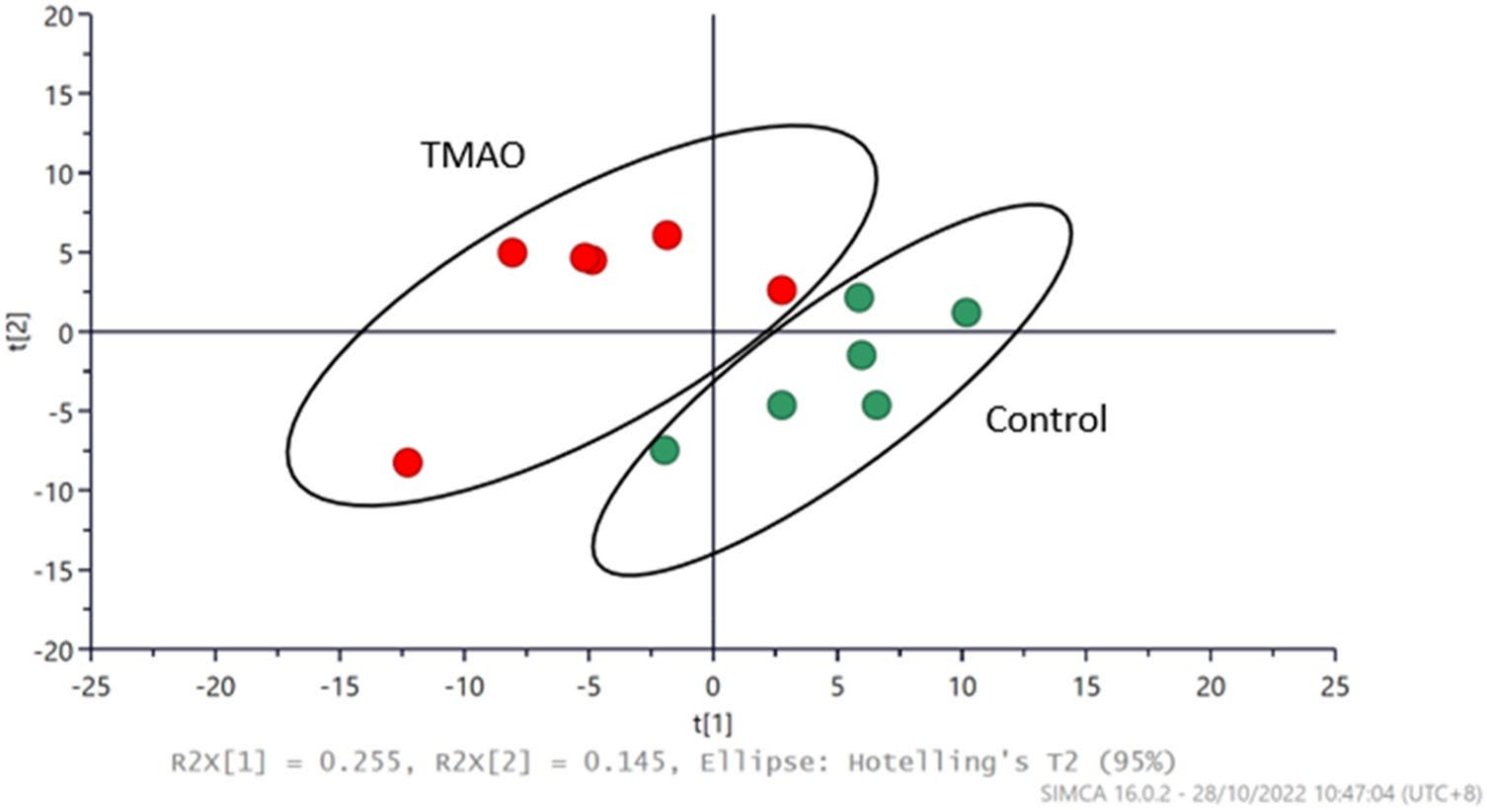
Metabolic profiling of untreated vs. TMAO-treated HMEC-1 at 48H after LC/MS analysis. The Principal Component Analysis (PCA) score plot shows the distinctiveness of metabolic profiles among untreated (Control) and TMAO-treated in HMEC-1 treated groups (*n* = 6).

Oxidative stress and inflammation are well-documented to cause changes in lipid profiles which contributes to increased production of high fat/cholesterol-mediated endothelial dysfunction ^27^. Given that pronounced inflammation and oxidative stress were observed after 48H of TMAO treatment, metabolite profiling of several lipid markers was analysed at this time point (Table 1). The principal component analysis (PCA) score plot showed distinctive metabolite profiles between the control and TMAO-treated groups, indicating a marked overall modification of metabolites after 48H of treatment in ECs (Fig. 7). The variable importance in projection (VIP) scores estimate the importance of each metabolite in the orthogonal projection to latent structure discriminant analysis (OPLS-DA) model; hence a VIP value close to 1 or higher than 1 highlights an important metabolite in the model (Table 1). It was observed that arachidonic acid, and especially its associated metabolites (prostaglandins), as well as palmitic acid, were enriched. This suggests that these metabolites were key contributors towards the separation between the control and TMAO-treated groups (Fig. 7, Table 1). Furthermore, TMAO treatment showed limited or no changes to choline and betaine, while acetylcarnitine appeared to be enriched. However, it is important to note that without cross-validating the OPLS-DA model, the accuracy of the VIP analysis regarding the contribution of metabolites remains unclear, hence standard statistical analysis was performed to identify the key metabolites that were altered by TMAO treatment. Specifically, only the levels of the bile acid (BA) metabolites (glycodeoxycholic acid) and prostaglandin B2 (PGB2) were significantly (*P* < 0.05) increased by TMAO treatment.

**Table 1:** Metabolites identified by LC/MS analysis in HMEC-1 after 48H of TMAO treatment as compared to untreated cells (Control).

Retention Time/mn	m/z	Identified Compounds	Normalised Peak Intensity (%)		
			Control	TMAO	VIP values
0.78	104	Choline	0.0145 ± 0.0105	0.0197 ± 0.0129	0.716
0.8	118	Betaine	0.000891 ± 0.000589	0.000904 ± 0.000936	0.72
0.83	204	Acetylcarnitine	0.000714 ± 0.000201	0.000999 ± 0.000319	0.989
5.779	464	Glycocholic acid	0.0000677 ± 0.0000522	0.000122 ± 0.0000926	0.99
5.879	353	Prostaglandin E1 (PGE1)	0.000353 ± 0.000108	0.000513 ± 0.000170	1.217
5.94	351	Prostaglandin D2 (PGD2)	0.000503 ± 0.000106	0.000590 ± 0.000168	1.147
6.717	333	Prostaglandin A1 (PGA1)	0.000580 ± 0.000228	0.0010495 ± 0.000318*	1.298
6.75	448	Glycodeoxycholic acid	0.000233 ± 0.0000603	0.000352 ± 0.0000783*	0.821
6.784	333	Prostaglandin B2 (PGB2)	0.000460 ± 0.000118	0.000664 ± 0.0004293	0.837
7.889	391	Deoxycholic acid (DCA)	0.000948 ± 0.000396	0.00162 ± 0.00213	1.164
10.77	303	Arachidonic acid (AA)	0.000835 ± 0.000449	0.00153 ± 0.000638	0.848
10.85	524	LPC C18:0 (lysophosphatidylcholine)	0.0121 ± 0.008	0.0178 ± 0.00948	0.717
11.44	255	Palmitic acid	0.00194 ± 0.000650	0.00302 ± 0.00135	1.054
11.44	522	LPC C18:1	0.00619 ± 0.00167	0.00546 ± 0.00131	0.668

Values are represented as mean ± SD, *n* = 6, *Significantly different from the control (*P* < 0.05, Student’s unpaired *t*-test).

## Discussion

**Figure 8:**
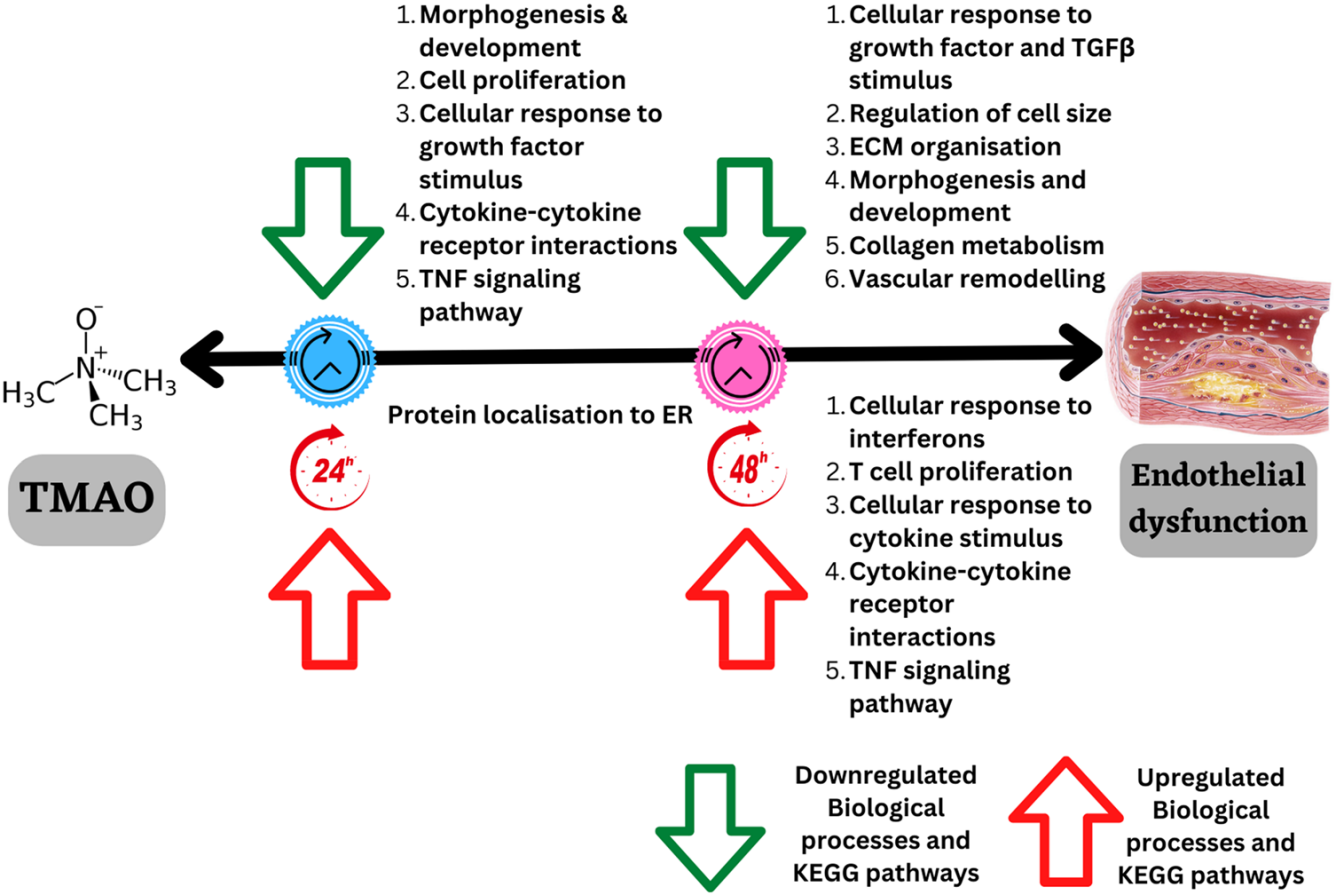
Summary of time-dependent distinct molecular signatures induced by TMAO in HMEC-1 after 24H and 48H of treatment.

Our study demonstrates that TMAO treatment is associated with chronologically distinct molecular signatures in HMEC-1, especially TNF signalling pathway and inflammation, as well as structural remodelling. Specifically, genes involved in morphogenesis and development, response to growth factors stimulus, as well as in the response to the pro-inflammatory molecule TNF, were downregulated after 24H of TMAO treatment. At 48H, genes involved in morphogenesis and development were still downregulated, as well as growth factors. In addition, genes involved the response to TGF-β, as well as in ECM modelling and collagen metabolism were specifically downregulated at 48H. By contrast, genes linked to cellular response to interferons, cytokine production, T cell proliferation, cytokine stimulus, and TNF signalling pathways were specifically upregulated at 48H (Fig. 8), highlighting an important inflammatory phenotype induced by TMAO at this time-point. The inflammatory cytokine production is known to be mediated through the NOD-like receptor signalling pathway, which triggers NF-kB, type I interferon (IFN), and MAPK signalling pathways, which eventually controls the release of inflammatory molecules such as IL-6 and TNFα ^28^. Consistent with this, after 48H of treatment, we observed activation the NOD-like receptor signalling, cytokine-cytokine receptor interaction, NF-kappa B, Janus kinase-signal transducer and activator of transcription (JAK-STAT) pathways (Fig. 3). Interestingly, the MAPK signalling pathway is also known to trigger vasoconstriction, which increases cardiac and vascular remodelling, activating endothelial dysfunction and resulting in atherosclerosis^29^. The MAPK signalling pathway could therefore participate in TMAO effects at disrupting endothelial function.

Inflammation is usually accompanied by oxidative stress which was validated here through the measurement of ROS produced after TMAO treatment at both 24H and 48H. This observation was consistent with several other studies showing an increase in ROS and inflammatory biomarkers in HUVEC, HAEC, and HVSMC after 6H of TMAO treatment (100, and 200 µM)^16,30^. This increase in oxidative stress could participate in the lower cell viability observed in our experiments. Indeed, a reduction of the number of viable cells (that may be due to cell death and/or loss of proliferation) was observed for HMEC-1 at both 24H and 48H, consistent with downregulation of the cellular response to growth factors and cell proliferation pathways observed in our global analyses. Our findings on viability are consistent with several other studies demonstrating that TMAO induces a reduction in cell viability^19,31^, while in contrast, some studies reported that TMAO does not lower cell viability^20,30,32,33^. While these discrepancies may be attributed to different cell types or TMAO concentrations, we show here that the duration of TMAO treatment is an important factor that, combined with others such as metabolic background for instance, may induce differential results.

After of 48H treatment, our global analyses data showed that TMAO also caused modulations of genes involved in ECM organisation, morphogenesis and development, collagen metabolic processes, cell adhesion and neuroactive ligand-receptor interaction. Interestingly, the neuroactive ligand-receptor interaction pathway is closely linked to the development and progression of cardiovascular diseases^34^, while ECM remodelling contributes to vascular deterioration causing tissue repair and affecting angiogenesis^35^. Consistent with this observation, dose-dependent reduction in HUVEC-ECM adhesion reduced by 0.92 ± 0.08-fold compared with control after 100 µM TMAO treatment for 24H ^30^. These findings were confirmed by RT-qPCR analysis here using biomarkers for ECM and fibrosis (CTGF, TGF-β1, MMP2), known to trigger structural changes in ECs. While TGF-β1 itself did not show any significant changes with TMAO treatment, the CTGF gene expression initially decreased after 24H but increased after 48H. Conversely, RT-qPCR results showed that the relative gene expression level of MMP2 was significantly higher than the control at 24H, but no significant changes were observed for 48H. These findings suggest that ECM remodelling occurs in a time-dependent manner in HMEC-1, with MMP2 being activated first while CTGF activation would occur later. This gene regulations may have consequences on the suppression of biological processes such as collagen metabolism and ECM organisation processes/pathways identified in our global analyses. In addition, ECM remodelling is known to trigger various other cellular responses such as proliferation, migration, cell fate decisions, and cell death^36–38^, that could therefore play roles in TMAO-induced endothelial phenotypes. While some of the key pathways such as inflammation or ECM organisation processes identified through transcriptomics approach were verified using qPCR, additional phenotypic experiments such as cell adhesion assay or gelatinase assay could be performed to strengthen the conclusion of the current study.

In support of the transcriptomic changes, LC/MS data showed that BAs and arachidonic acid metabolites increased after TMAO treatment. BAs are a class of cholesterol-derived hormones which facilitate the digestion and absorption of dietary lipids. Their functions include systemic endocrine regulation, lipid, and glucose metabolism^39,40^. In addition, BAs are known to induce the expression of adhesion molecules in ECs by triggering oxidative stress, and through the activation of NF-kB and p53 signalling pathways^39^. Metabolites from arachidonic acid (essential fatty acids), including prostaglandins, thromboxanes and leukotrienes, were reported to have pro-inflammatory effects, resulting in a vasoconstricting phenotype of  the endothelium^41,42^. Prostaglandins are lipid autacoids which are produced at the location of tissue damage and are also involved in inflammation, blood flow, and development of blood clots. This suggests that the modulation of BAs and arachidonic acid metabolite levels by TMAO are closely linked to inflammation and may contribute to the initiation of early stages of endothelial dysfunction^43^.

In conclusion, using global transcriptomic approaches, our study suggested that TMAO induces time-dependent changes in HMEC-1, particularly structural changes, TNF-induced modifications, and inflammatory phenotypes over the early (24H) and late (48H) treatment durations. While TNF and inflammation pathways are first repressed (24H), they become activated at later stages (48H). Structural modification pathways show a more complex pattern with collagen-related pathways possibly repressed at both 24H and 48H, while other ECM pathways, involving MMP2 or CTGF, only appear activated at 24H and 48H, respectively. In contrast, common biological processes downregulated for both treatment durations include cellular response to growth factors as well as morphogenesis and development, suggesting consistent modification of proliferation and/or cell death induced by TMAO during the treatment duration. While our results proposed several molecular pathways, these findings would need to be validated in future studies using in vivo animal models or in clinical setting in order to identify new targets for therapeutics to alleviate cardiovascular complications associated with TMAO in the right intervention window.

## Materials and methods

### Cell culture

Human dermal microvascular endothelial cells (HMEC-1), obtained from American Type Culture Collection (ATCC, CRL-3243, Manassas, VA, USA) were cultured as previously described^44^. Briefly, HMEC-1 were maintained at 37 °C with 5% CO_2_ in MCDB-131 media, supplemented with 20% foetal bovine serum (FBS), 0.001% recombinant human epidermal growth factor (10 ng/ml), 1% penicillin–streptomycin, and 200 mM L-glutamine. TMAO, (CH_3_)_3_N(O) was purchased from Sigma-Aldrich (Singapore, Cat #317,594). During TMAO treatment (50–250 µM), the cells were cultured in serum-starved media (2% FBS) for 24H or 48H. After treatment, they were harvested and subjected to cell viability assays, ROS assays, or RNA extraction for gene sequencing and RT-qPCR analysis. For metabolite profiling, cells were treated with TMAO (50 µM) for 48H.

### Cell viability assay

Cell viability assays were performed using the PrestoBlue reagent (Thermo Scientific, Singapore) as per manufacturer’s recommendations. Briefly, after 24H or 48H of TMAO treatment, PrestoBlue reagent was added to the cells for 10 min in technical triplicates. After incubation, excitation and emission wavelengths were measured for each well at 570 nm and 590 nm, respectively, using a Varioskan LUX multimode microplate reader (Thermo Scientific, Singapore). Percentage of cell viability was normalised to control.

### RNA extraction and reverse transcription

HMEC-1 were lysed, and total RNA was extracted using the Aurum™ Total RNA Mini Kit (Bio-Rad Laboratories, Hercules, CA, USA), as previously described^45,46^. RNA quality and concentration were determined using NanoDrop One Microvolume UV–Vis Spectrophotometer (Bio-Rad Laboratories, USA). RNA samples (50 ng/µL) were reverse transcribed into cDNA by the T100™ Thermal cycler (Bio-Rad laboratories, USA) using Bio-Rad iScript cDNA Synthesis Kit according to manufacturer’s recommendations.

### RNA sequencing

Total RNA from treated HMEC-1 was used to construct libraries using the VAHTS® Universal V8 RNA-seq Library Prep Kit for Illumina NR605 and sequenced using Illumina NovaSeq6000 S4 flowcell PE150 sequencer at Azenta Life Sciences (Hangzhou, China). Raw FASTQ reads were checked for quality and processed using the nf-core RNAseq bioinformatics pipeline ^47^ version 3.6 to generate the read counts for downstream analyses.

### Downstream analyses of RNA sequencing data

DEG data for the 24H vs. control and 48H vs. control were generated using DESeq2 package^48^. Genes with at least a 1.4-fold change and a false discovery rate (FDR) < 0.05 were considered significant. Gene set enrichment analysis (GSEA) was performed on the fold-change ranked gene lists using the ClusterProfiler package with the Gene Ontology Biological Process (GO BP) and Kyoto Encyclopedia of Genes and Genomes (KEGG) pathways^49^. A tree map summary of the enriched GO BP terms was generated using Revigo^50^. Variance Stabilizing Transformation (VST) normalized expression was used for visualizing the expression levels.

### ROS quantification

Intracellular ROS production in HMEC-1 was analysed using 10 µM of 2’,7’-Dichlorofluorescin diacetate (DCFDA, Cayman Chemical, USA) as described earlier^51,52^. Briefly, after 24H or 48H of TMAO treatment, HMEC-1 cells were incubated with DCFDA for 1 h. Subsequently, DCFDA was removed by rinsing with Hank’s Balanced Salt Solution (HBSS, Grand Island IsleChem, USA), and the cells were counter-stained with Hoechst 33342 (Pierce Biotechnology, USA) for 15 min at 37 °C. Cells were rinsed again with HBSS and the fluorescence intensity was measured at the excitation and emission wavelengths of DCFDA (Ex/Em = 485/520 nm) and Hoechst (Ex/Em = 350/461 nm). Readings were taken using a Varioskan LUX multimode microplate reader (Thermo Scientific, Singapore). DCFDA values were normalised to Hoechst readings and results were averaged and expressed as fold changes of control.

### Real-time quantitative polymerase chain reaction (RT-qPCR)

The comparative cycle threshold (2^−ΔΔCt^) method of RT-qPCR was conducted according to earlier reports ^53^. The CFX96 Real-time PCR system (Bio-rad, USA) was used to evaluate the relative gene expression of IL-6, chemoattractant protein 1, Monocyte Chemoattractant Protein-1 (MCP-1)/CCL2), EDN1, CTGF, MMP2, TGF-β1 ICAM and IL-1β (Supplementary Table 1). RT-qPCR was performed in 96-well plates with 10 µL volume reactions per well (10 µM primers and SYBR Green master mix (Bio-rad, USA) prepared in triplicates). Glyceraldehyde-3-phosphate dehydrogenase (GAPDH) was used as the reference housekeeping gene. Relative gene expression was calculated using the 2^-ΔΔCT^ method, where gene expression changes were expressed as fold changes of control.

### Lipid extraction and metabolite profiling of HMEC-1 using liquid chromatography-mass spectrometry (LC/MS)

Chloroform:methanol method was used to extract lipids and LC/MS was carried out with minor modifications as previously reported^54–56^. Briefly, the extracts were vortexed and centrifuged at 10,000 rpm for 5 min. The supernatant was transferred into a 1.5 mL tube and dried using a vapor centrifuge at 45 ºC for 30 min. Next, the dried samples were reconstituted in methanol (200 µL) before being subjected to LC/MS (Shimadzu LC/MS-8050) analysis using a C18 reverse phase HPLC column (Zorbax SB-C18- 3.5 microns, 2.1 × 100 mm). A gradient elution was performed, which involved a mobile phase (A) with 0.1% of formic acid in water, and (B) with 0.1% of formic acid in acetonitrile. Data analysis was performed using a targeted approach based on the measured peak intensity obtained for each sample. The peak intensities corresponding to various molecular weights (m/z) were normalised to the total peak intensity within each sample, to account for the differences of concentrations. Subsequently, normalised data for each sample was analysed using PCA and OPLS-DA scores plot based on the selected peaks. Based on the comparison of the retention time, m/z and MSMS with reference standards and comparison of MSMS spectra with reference database (https://hmdb.ca/), certain fatty acids, lipids, and other small molecules were identified, consistent with our earlier work^57–59^.

### Statistical analyses

Experimental data for cell viability, ROS assays, and RT-qPCR were plotted using GraphPad Prism 6 (GraphPad, San Diego, CA, USA). PCA, OPLS-DA plots, and index values of the VIP in OPLS-DA were used to calculate the importance of individual metabolite features in spectra generated by the Soft Independent Modelling by Class Analogy (SIMCA) software^60^. Experimental data were obtained from at least three independent experiments and are represented as either mean ± SEM or mean ± SD (LC/MS), as appropriate. Group mean values were analysed by unpaired Student’s *t*-test or one-way ANOVA with post hoc analysis using Dunnett’s test, as appropriate. Statistical significance was considered when the *p*-value was < 0.05.

### Supplementary Information

Supplementary Information 1.

Supplementary Information 2.

## Data Availability

The datasets generated and/or analysed during the current study are available in the Gene Expression Omnibus (GEO) repository, GSE235204.

## Abbreviations

CVDs: Cardiovascular diseases
TMAO: Trimethylamine N-oxide
ECs: Endothelial cells
HMEC-1: Human microvascular endothelial cells
DEG: Differentially expressed gene
RT-qPCR: Real-time quantitative polymerase chain reaction
TMA: Trimethylamine
FMO: Flavin monooxygenase
ROS: Reactive oxygen species
GO: Gene ontology
BP: Biological process
KEGG: Kyoto encyclopedia of genes and genomes
DCFDA: Dichlorofluorescin diacetate
NES: Normalised enrichment score
DUSP1: Dual specificity phosphatase 1
AP-1: Activator protein 1
IL-6: Interleukin 6
CCL2: Chemoattractant protein 1
EDN1: Endothelin 1
CTGF: Connective tissue growth factor
MMP2: Matrix metallopeptidase 2
TGF-β1: Transforming growth factor beta 1
ICAM: Intracellular adhesion molecule 1
IL-1β: Interleukin 1 beta
GAPDH: Glyceraldehyde-3-phosphate dehydrogenase
JAK-STAT: Janus kinase-signal transducer and activator of transcription
PCA: Principal component analysis
OPLS-DA: Orthogonal projection to latent structure discriminant analysis
VIP: Variable importance in projection
SIMCA: Soft independent modelling by class analogy
TNF: Tumour necrosis factor
MAPK: Mitogen-activated protein kinase
IFN: Type I interferon
NOD: Nucleotide oligomerization domain
ECM: Extracellular matrix
NF-kB: Nuclear factor kappa B
