# Supplementary material for: Time-dependent specific molecular signatures of inflammation and remodelling are associated with trimethylamine-N-oxide (TMAO)-induced endothelial cell dysfunction
Source: Sci Rep. 2023 Nov 20;13:20303. doi: 10.1038/s41598-023-46820-7 (PMC10661905; doi:10.1038/s41598-023-46820-7)
Supplement: Supplementary file 1 — Supplementary Information 1. [file 41598_2023_46820_MOESM1_ESM.docx]

**Time-dependent specific molecular signatures of inflammation and remodelling are associated with Trimethylamine-N-oxide (TMAO)-induced endothelial cell dysfunction.**

Meyammai Shanmugham^1,3^, Arun George Devasia^1^, Yu Ling Chin^1^, Kang Hao Cheong^1^, Eng Shi Ong^1^, Sophie Bellanger^3^, Adaikalavan Ramasamy^2^, Chen Huei Leo^1^

^1^Science, Math & Technology, Singapore University of Technology & Design, Singapore 487372

*^2^*Genome Institute of Singapore, Agency for Science, Technology and Research, Singapore

^3^A*STAR Skin Research Labs, Agency for Science, Technology and Research, Singapore

**Corresponding Author:**

Dr Chen Huei Leo

Singapore University of Technology and Design,

8 Somapah Road, Singapore 487372, Republic of Singapore

E-mail address: chenhuei_leo@sutd.edu.sg

Tel.: +65 6434 8213

**
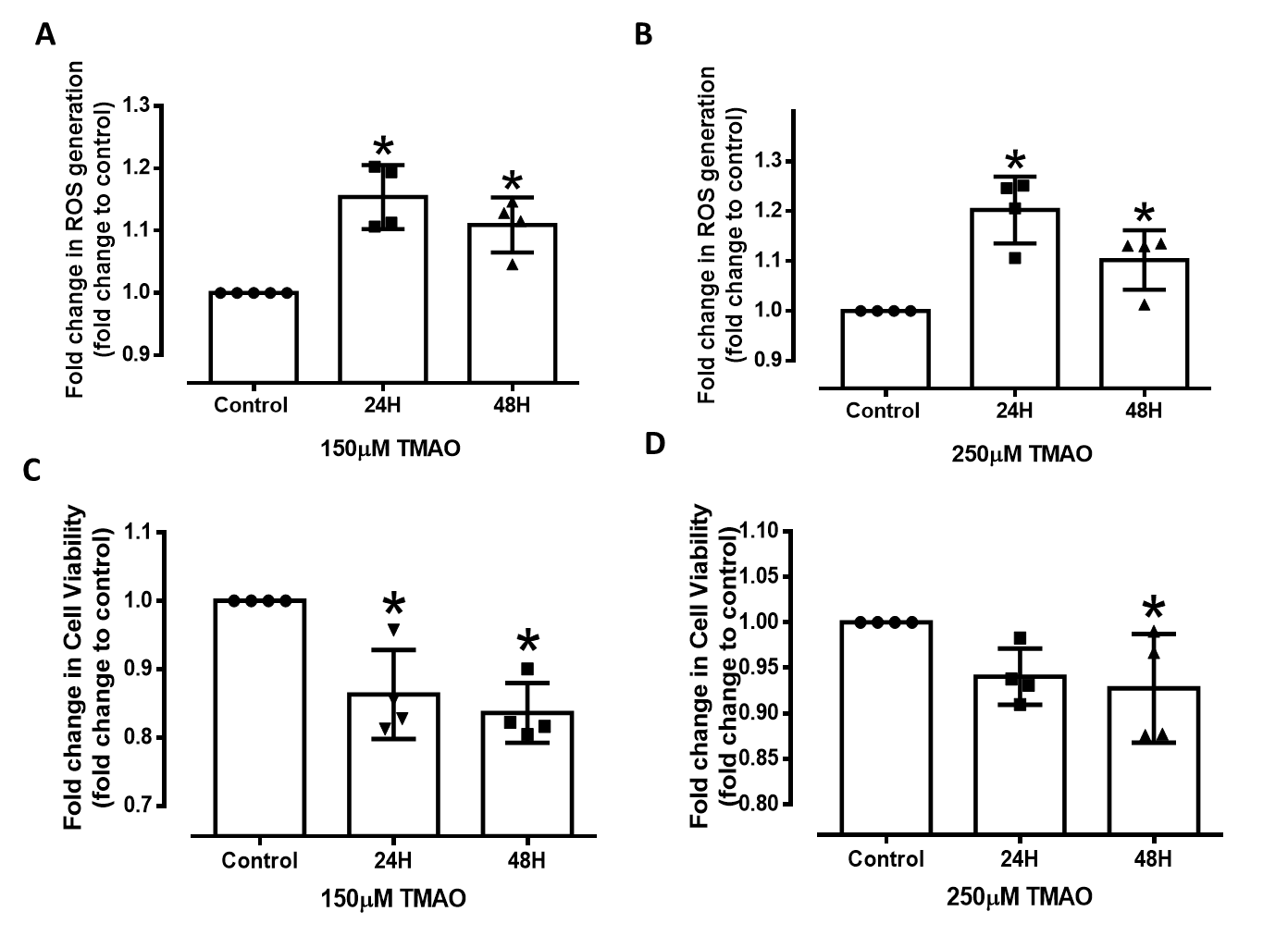
**

**Supplementary Figure 1: TMAO induces ROS production and lowers cell viability in HMEC-1 cells.** (A) and (B) ROS generation after treatment with 150 µM and 250 µM of TMAO, respectively. (C) and (D) Quantification of cell viability after treatment with 150 µM and 250 µM of TMAO, respectively. Values are expressed as mean ± SEM; the number of experiments per group is shown by individual data points. *Significantly different to control (P < 0.05, One-way ANOVA, Dunnett’s test).


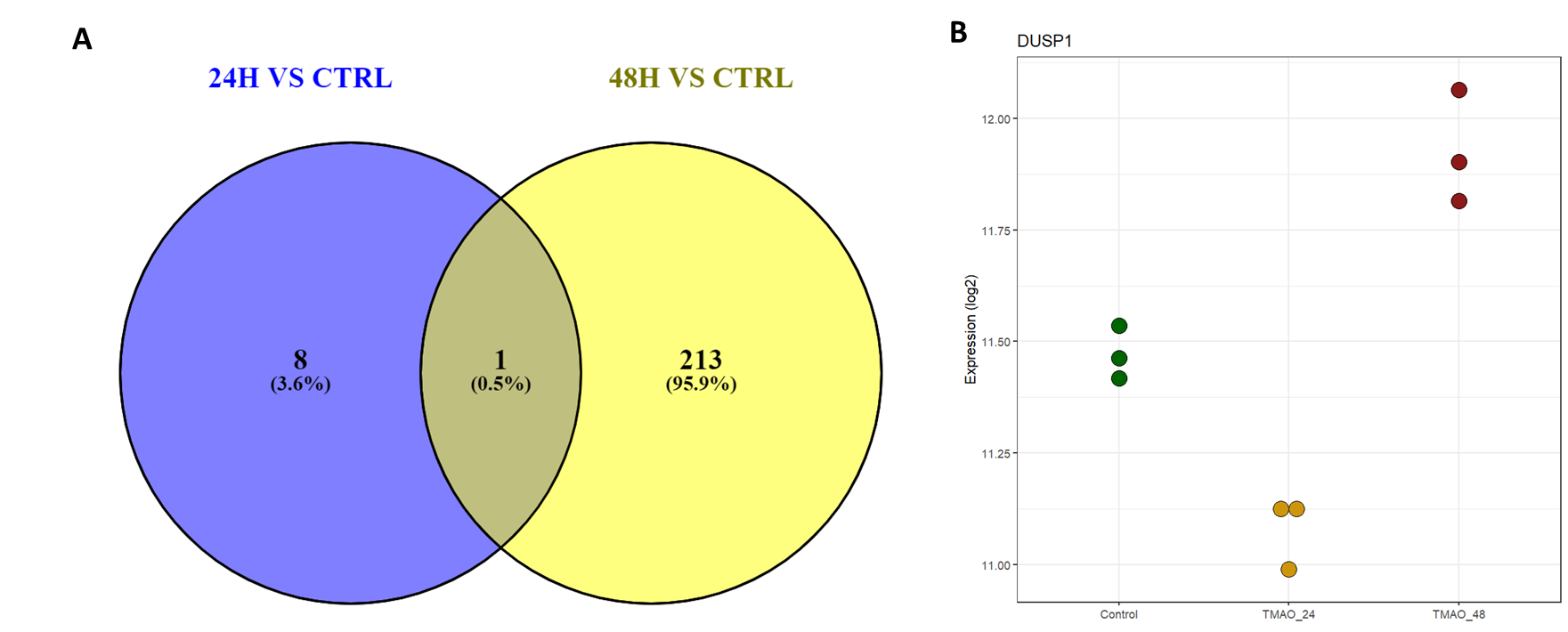


**Supplementary Figure 2: Transcriptomic profiling of HMEC-1 treated with TMAO for 24H and 48H**. (A) Venn diagram showing the number of significant genes (1.4-fold change and FDR < 0.05) modulated by TMAO at 24H vs. control (blue), and at 48H vs control (yellow). DEGs were identified using the p-value < 0.05 and log_2_FC > = |0.4| criteria. (B) VST normalized expression levels of DUSP1 showing this gene to be downregulated at 24H analysis and upregulated at 48H.


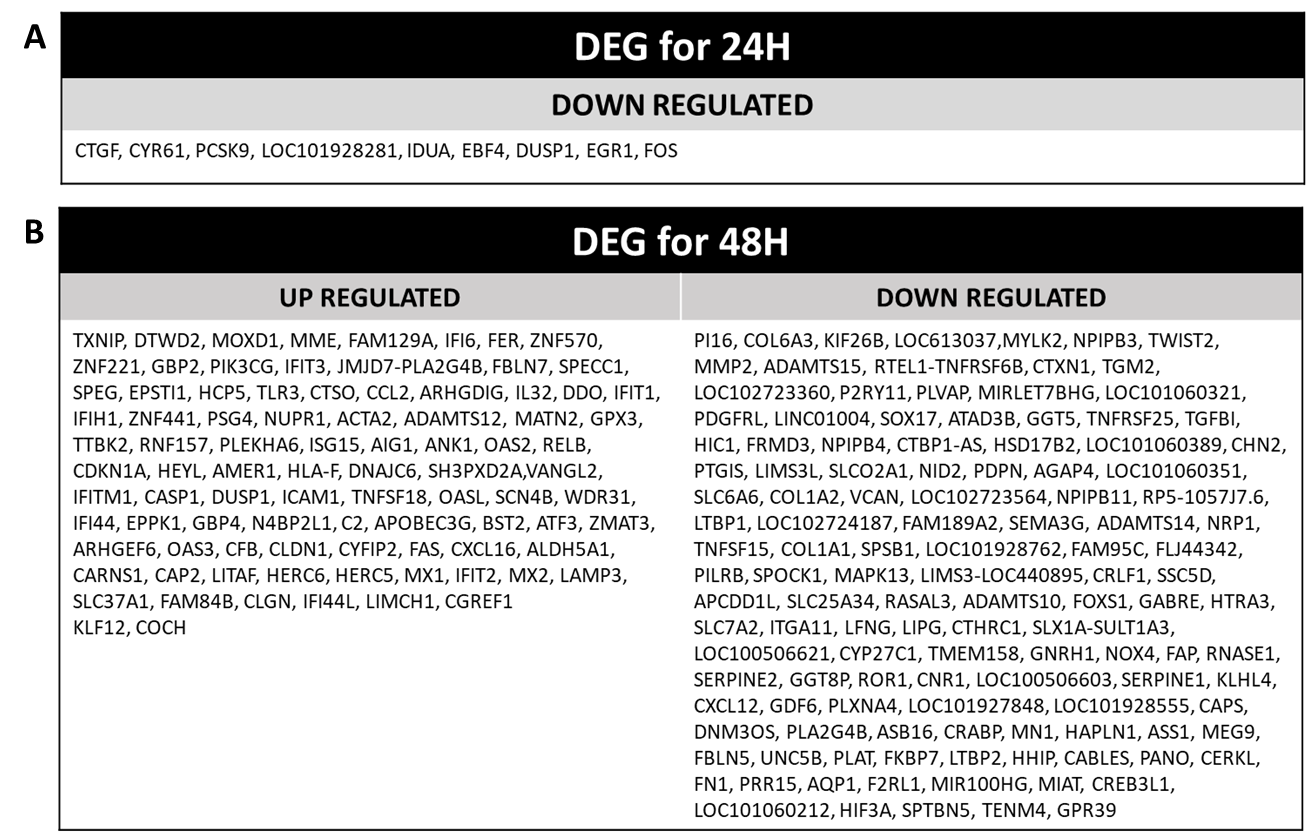


**Supplementary Figure 3: DEG results showing genes modulated in HMEC-1 treated with TMAO for 24H and 48H**. (A) Genes modulated at 24H (all downregulated). (B) Genes modulated at 48H: 90 genes were upregulated; 124 genes were downregulated. DEGs were identified using the p-value < 0.05 and log_2_FC > = |0.4| criteria.

**
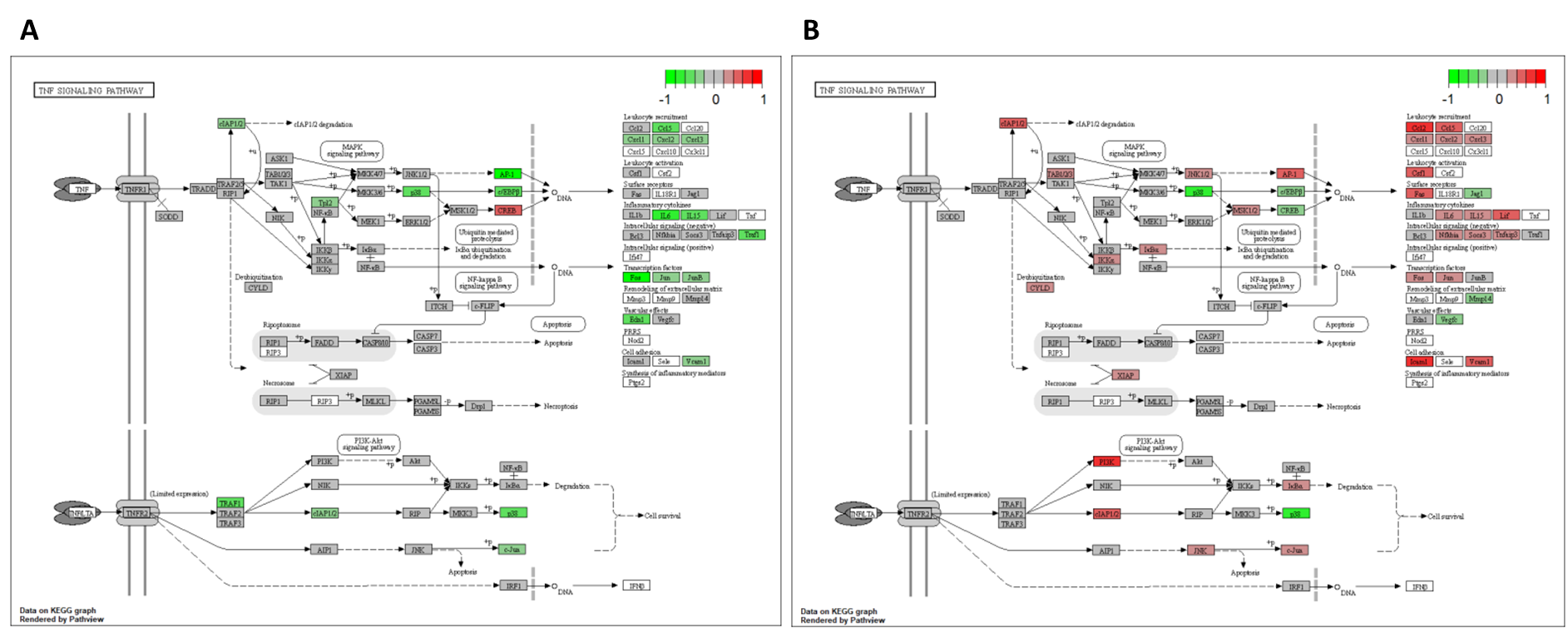
**

**Supplementary Figure 4: Mapping of genes modulated by TMAO in the “TNF signalling pathway” at 24H and 48H**. (A) “TNF signalling pathway” identified as downregulated via KEGG after 24H of TMAO treatment (50 µM). (B) “TNF signalling pathway” identified as upregulated after 48H of TMAO treatment (50 µM). The gene hits highlighted in red are activated compared to control while the gene hits highlighted in green are repressed. All the other genes related to the pathway are also shown.

**
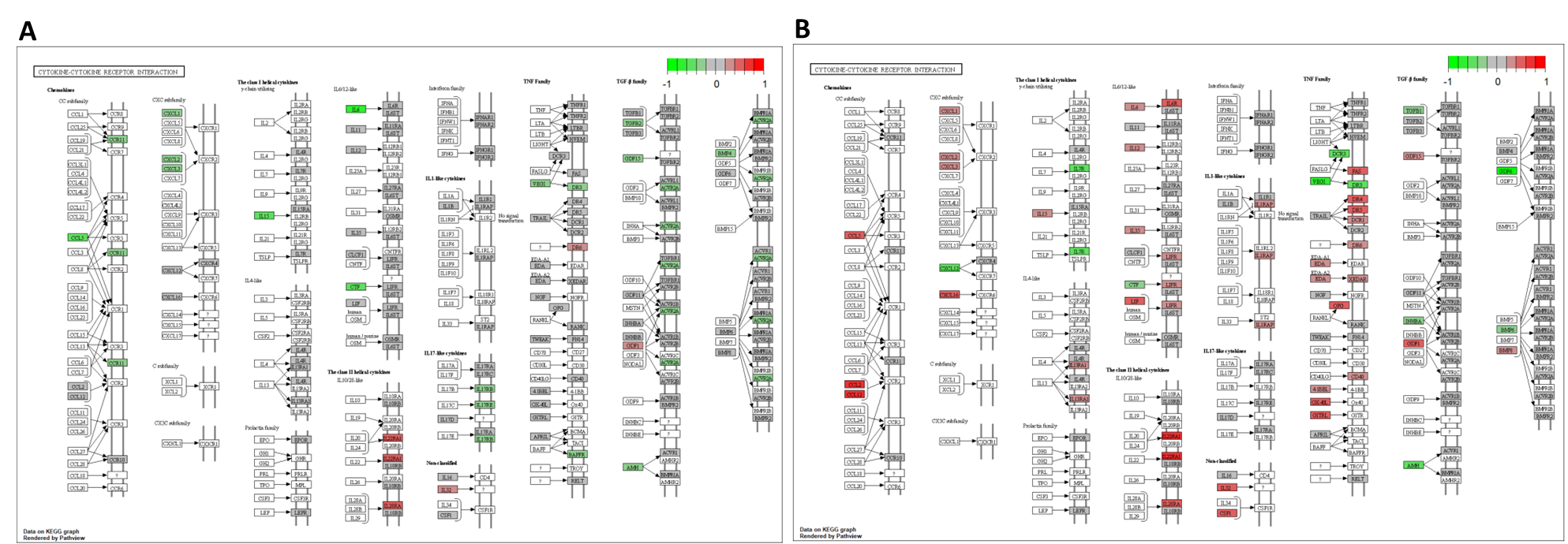
**

**Supplementary Figure 5: Mapping of genes modulated by TMAO in the “cytokine-cytokine receptor interaction” at 24H and 48H.** (A) “Cytokine-cytokine receptor interaction” identified as downregulated via KEGG after 24H of TMAO treatment (50 µM). (B) “Cytokine-cytokine receptor interaction” identified as upregulated after 48H of TMAO treatment (50 µM). The gene hits highlighted in red are activated compared to control while the gene hits highlighted in green are repressed. All the other genes related to the pathway are also shown.

**Supplementary Table 1: List of RT-qPCR primers used for this study**

| **Gene** | **Forward primer sequence (5’-3’)** | **Reverse primer sequence (5’-3’)** |
| --- | --- | --- |
| GAPDH | ACAGTTGCCATGTAGACC | TTGAGCACAGGGTACTTTA |
| IL6 | GCAGAAAAAGGCAAAGAATC | CTACATTTGCCGAAGAGC |
| CCL2 | AGACTAACCCAGAAACATCC | ATTGATTGCATCTGGCTG |
| EDN1 | CAAGCAGGAAAAGAACTCAG | CTGGTTTGTCTTAGGTGTTC |
| CTGF | TTAAGAAGGGCAAAAAGTGC | CATACTCCACAGAATTTAGCTC |
| MMP2 | TTCTGGAGATACAATGAGGTG | CTTGAAGAAGTAGCTGTGAC |
| TGFBI | CCCACAACGAAATCTATGAC | TGTATTTCTGGTACAGCTCC |
| ICAM1 | ACCATCTACAGCTTTCCG | TCACACTTCACTGTCACC |
| IL1B | CTAAACAGATGAAGTGCTCC | GGTCATTCTCCTGGAAGG |
